# Circular RNAs hsa_circ_0032462, hsa_circ_0028173, hsa_circ_0005909 are predicted to promote CADM1 expression by functioning as miRNAs sponge in human osteosarcoma

**DOI:** 10.1371/journal.pone.0202896

**Published:** 2018-08-28

**Authors:** Gaoyang Chen, Qingyu Wang, Qiwei Yang, Zhaoyan Li, Zhenwu Du, Ming Ren, Haiyue Zhao, Yang Song, Guizhen Zhang

**Affiliations:** 1 Department of Orthopedics of the Second Hospital of Jilin University, Changchun, Jilin, China; 2 Research Centre of the Second Hospital of Jilin University, Changchun, Jilin, China; 3 The Engineering Research Centre of Molecular Diagnosis and Cell Treatment for Metabolic Bone Diseases of Jilin Province, Changchun, Jilin, China; University of Toronto, CANADA

## Abstract

**Background:**

Osteosarcoma (OS) is a primary malignant bone tumor with a high fatality rate. Many circRNAs have been proved to play important roles in the pathogenesis of some diseases. However, the occurrence of circRNAs in OS remains little known.

**Methods:**

The circular RNA (circRNA) expression file GSE96964 dataset, which included seven osteosarcoma cell lines and one control sample (osteoblast cell line), was downloaded from the Gene Expression Omnibus (GEO) database to explore the potential function of circRNAs in osteosarcoma by competing endogenous RNA (ceRNA) analysis. Three gene expression profiles of OS were downloaded from GEO database and then used for the pathway enrichment analysis, Venn analysis and protein-protein interaction (PPI) network analysis. Real-time qPCR validation and RNA interference were conducted to verify our prediction.

**Results:**

Differentially expressed circRNAs between OS and control, including 8 up-regulated and 102 down-regulated circRNAs, were generated and ceRNA analysis for 5 most up-regulated or 5 most down-regulated circRNAs in OS were then performed. The pathway enrichment analysis of gene expression profiles indicated differentially expressed genes (DEGs) of three gene profiles significantly enriched in cell cycle pathway, cell adhesion molecules (CAMs) pathway, oxidative phosphorylation pathway, cytokine-cytokine receptor interaction pathway, p53 signaling pathway and proteoglycans in cancer pathway, which were critical important pathways in the pathogenesis of OS. The Venn analysis showed that 2 (one is a pseudogene) up-regulated and 39 down-regulated DEGs were co-expressed in all three gene profiles. Then PPI networks of 41 co-expressed DEGs (up- and down-regulated DEGs) were constructed to predict their functions using the GeneMANIA. The expression levels of these related RNAs also matched our predictions really well.

**Conclusion:**

Ultimately, we found cell adhesion molecule 1 (CADM1) gene was not only a co-expression mRNA of the three mRNA expression profiles of OS, but also are predicted to be regulated by hsa_circ_0032462, hsa_circ_0028173, hsa_circ_0005909 by functioning as miRNAs ‘Sponge’ in human osteosarcoma. These over-expressed circRNAs may result in the over expression of CADM1 which promote the development of OS. We envision this discovery of these important moleculars, incuding hsa_circ_0032462, hsa_circ_0028173, hsa_circ_0005909 and CADM1 may lead to further development of new concepts, thus allowing for more opportunities in diagnosis and therapy of OS.

## Introduction

Osteosarcoma (OS) is one of the most common primary malignancies in children, adolescents and young adults with a frequency increasing by 0.3% per year [1]. The tumor is a primary malignant bone tumor originating from mesenchymal cells of bone [2]. The high degree of malignancy results in a high fatality rate. Recently, surgery with chemotherapy is the first-line treatment for most OS [3]. However, the prognosis for patients with metastatic OS is poor with 5-year survival of no more than 20% [4]. Multiple associations have been made between the development of OS and race, gender, age, various genomic alterations, exposure situations among others [5]. Nevertheless, the origin and etiology of OS is further complicated for its extreme rearranged genome, high genetic instability, and lack of precursor lesions [6].

Circular RNA (circRNA) is a class of noncoding RNA which was discovered decades ago. Accumulating evidence has suggested that circRNAs play important roles in oncogenesis and tumor progression [7]. Pandolfi et al. presented a hypothesis called the competing endogenous RNA (ceRNA) mechanism, which proposed that transcripts such as mRNAs, pseudogenes and lncRNAs can serve as natural miRNA sponges by competitive binding to miRNA response elements (MREs) to suppress their expression and function [8]. Previous studies have well shown that circRNA function through a ceRNA mechanism [9]. More interestingly, Legnini et al. demonstrated that endogenous circRNAs might generate proteins, thereby expanding the eukaryotic proteome and revealing novel modes of cap-independent translation [10]. Recent years, circRNAs were found to play roles in different types of cancer and could potentially be powerful biomarkers for cancer for their long half-lives and resistance to common degradation pathways[11]. The circRNA hsa_circ_0001564 was found to act as miR-29c-3p sponge to mediate the tumorigenicity in OS [12]. Deng *et al*. also suggested that circ_0009910 could act as a sponge of miR-449a and upregulated miR-449a functional target IL6R in their OS study [13]. These findings indicated that circRNAs could play critical roles in the pathogenesis of OS, but more evidences need to be provided.

In order to further investigate differentially expressed circRNAs and their molecular biological roles in OS. One circRNA expression profile (GSE96964) and three gene expression profiles (GSE42352, GSE33382, GSE36001) of OS were downloaded from the Gene Expression Omnibus (GEO) database of the National Center of Biotechnology Information (NCBI, http://www.ncbi.nlm.nih.gov/geo/). The significantly differentially expressed circRNAs between the OS and control samples may exhibit important function in OS development and progression. Furthermore, three gene expression profiles were also analyzed and their co-expressed differentially expressed genes (DEGs) were then found, which were thought to be with great significance in the pathogenesis of OS. The ceRNA mechanism of circRNAs showed cell adhesion molecule 1 (CADM1) gene, which was a co-expression DEG of the three gene expression profiles, was also in the ceRNA network of 5 most up-regulated circRNA. We conducted this study to explore potential roles of circRNAs in OS and the circRNAs-miRNAs-CADM1 regulatory way in OS was proposed for the first time.

## Materials and methods

### Microarray data

The date of circRNA microarray GSE96964 (GPL19978, Agilent-069978 Arraystar Human CircRNA microarray V1; Agilent Technologies Inc., California, USA) and three gene expression profiles were downloaded from GEO Profiles. A total of 8 samples (7 OS cell lines and 1 osteoblast cell line) were included in the circRNA microarray dataset. The gene expression profile of GSE42352 (GPL10295, Illumina human-6 v2.0 expression beadchip; Illumina Int., USA) contained 19 samples of OS cell lines and 3 samples of osteoblast cells lines. The gene expression profiles of GSE33382 (GPL10295, Illumina human-6 v2.0 expression beadchip; Illumina Int., San Diego, USA) contained 84 pre-treatment high-grade osteosarcoma diagnostic biopsies and 3 samples of osteoblast cells lines. The gene expression profiles of GSE36001 (GPL6102, Illumina human-6 v2.0 expression beadchip; Illumina Int., San Diego, USA) contained 19 samples of OS cell lines and 2 samples of osteoblast cells. (Table 1)

**Table 1:** The information of expression profiles.

	Series	Platforms	OS cell lines/ biopsies	Osteoblast cell lines
**circRNA profile**	GSE96964	GPL19978	7	1
**gene profile 1**	GSE42352	GPL10295	19	3
**gene profile 2**	GSE33382	GPL10295	84	3
**gene profile 3**	GSE36001	GPL6102	19	2

### Differential expression analysis

GEO2R (https://www.ncbi.nlm.nih.gov/geo/geo2r/) is an interactive online tool that allows users to compare two or more groups of samples in a GEO Series [14]. GEO2R was applied to detect differentially expressed circRNAs and genes between OS samples and osteoblast cell lines. The adjust P values were utilized to reduce the false positive rate using Benjamini and Hochberg false discovery rate method by default [14]. The adjust P < 0.05 and FC ≥ 2 were set as the cut off criterion. Then the significantly differentially expressed circRNAs and genes were found.

### The ceRNA analysis and pathway analysis of circRNA-related genes

We constructed circRNA-miRNA-target gene networks (5 most up-regulated circRNAs and 5 most down-regulated circRNAs) using Cytoscape to visualize their interactions based on the circRNA profile and gene profiles. In the network, we predicted the circRNA/miRNA interaction with miRNA target prediction tool (Circular RNA interactome) and target gene/miRNA interaction established by using TargetScan (www.targetscan.org) and miRDB (www.mirdb.org). The network was based on the theory of ceRNA mechanism that the circRNA shared the same miRNA with mRNA. Then the pathway analysis was performed for these up- or down-regulated circRNA-related genes using Database for Annotation, Visualization and Integrated Discovery (DAVID, https://david.ncifcrf.gov/) and OmicsBean (www.omicsbean.com) tools.

### Venn analysis

The significantly DEGs (P < 0.05 and FC ≥ 2) of the three gene expression profiles were imported into Functional Enrichment analysis tool (FUNRICH). Then the co-expression genes were acquired by using Venn analysis. To better understand the potential function of DEGs in OS, the up-regulated DEGs and down-regulated DEGs were analyzed separately.

### Pathway enrichment analysis

Kyoto Encyclopedia of Genes and Genomes (KEGG) is a collection of databases dealing with biological pathways, genomes, drugs, diseases, and chemical substances [15]. The Database for Annotation, Visualization and Integrated Discovery (DAVID, https://david.ncifcrf.gov/) is a web based online bioinformatics resource that aims to provide tools for the functional interpretation of large lists of genes or proteins[16]. The significantly DEGs (P < 0.05 and FC ≥ 2) of the three gene expression profiles were analyzed by DAVID tool separately. And also, the up-regulated DEGs and down-regulated DEGs were analyzed separately.

### PPI network construction

The protein-protein interaction (PPI) networks of co-expressed DEGs in three gene profiles were generated using the GeneMANIA (http://www.genemania.org/), which is a web-based database and a tool for the prediction of gene functions on the basis of multiple networks derived from different genomic or proteomic data/sources [16]. It is fast enough to predict gene functions with great accuracy using GeneMANIA because hundreds of datasets from BioGRID, GEO, I2D and Pathway Commons, as well as organism specific functional genomics data sets have been collected in this software.

### Cell culture and RNA extraction

The human osteosarcoma cell lines MG63, U2OS, 143B, HOS and the human osteoblast cell line hFOB1.19 were obtained from the American Type Culture Collection (ATCC). All cell lines were cultured in DMEM (Dulbecco minimum essential medium; Gibco, Shanghai, China) mixed with 10% FBS (fetal bovine serum) and 1% antibiotics (penicillin and streptomycin). Total RNA was extracted using TRIzol (Invitrogen) following the manufacturer’s introductions.

### Quantitative real-time PCR analysis and RNA interference

The circRNA and mRNA cDNAs were synthesized by reverse transcription from total RNA using a random priming method (PrimeScriptTMRT Reagent Kit; TaKaRa, Japan). The has-miR-142-5p and has-miR-388-3p cDNAs were synthesized from 2 μg of total RNA with an All-in-one miRNA First-Strand cDNA Synthesis (GeneCopoeia) Kit. Quantitative real-time PCR was performed using SYBR Green qPCR Master Mix (Thermo Fisher Scientific). The expression of hsa_circ_0032462, hsa_circ_0028173, hsa_circ_0005909 and CADM1 were normalized relative to the endogenous control of human glyceraldehyde-3-phosphate dehydrogenase (GAPDH) and the expression of has-miR-142-5p and has-miR-338-3p were normalized to U6 as an endogenous control. The relative expression was calculated using the 2−△△Ct method. All the primers were shown in Table 2.

**Table 2:** The primers of circRNAs and mRNA.

Gene	Primer sequences	
**hsa_circ_0032462**	**F:** **5’- TGAAACTGGATGAACAAGGGAG-3’**	**R:** **5’- GCCGTCTGTGCCAACAAC-3’**
**hsa_circ_0028173**	**F:** **5’- TCCGCTACCTCATCTCGT-3’**	**R:** **5’- GTTGCTACCACCACTCCC-3’**
**hsa_circ_0005909**	**F:** **5’- GTATCCACTTGCCCTTTA -3’**	**R:** **5’- TTACTCCAGCCTGTCTC -3’**
**CADM1**	**F:** **5’- CTGTGATTCAGCTACTGAATC -3’**	**R:** **5’- TAGAAAAATTCAGCAACTGAAAC -3’**
**GAPDH**	**F:** **5’- CGGACCAATACGACCAAATCCG-3’**	**R:** **5’- AGCCACATCGCTCAGACACC-3’**

Small interfering RNAs (siRNAs), which can target the back-splice junction of hsa_circ_0032462, hsa_circ_0028173 and hsa_circ_0005909 were designed and synthesized. The MG63 cells were transfected using GoldenTran-R (Golden Trans Technology, Changchun, China). The expression of related RNAs were calculated using the 2−ΔΔCt method.

### Data analysis

The bar graphs were constructed by Microsoft excel software. A value of P<0.05 indicated that the difference was statistically significant. Fold-changes of ⩾2 and P-values of <0.05 in circRNA and and three gene microarray data were regarded as significantly differentially expressed.

## Results

### Differentially expressed circRNA and genes

The differentially expressed circRNA and genes were generated by using GEO2R tool. Based on the thresholds of P < 0.05 and FC ≥ 2, a total of 110 differentially expressed circRNA were obtained in the osteosarcoma group compared to the control group, including 8 up-regulated and 102 down-regulated circRNAs. For the same thresholds, 991 DEGs including 456 up-regulated and 536 down-regulated DEGs were achieved from gene profile 1. 845 DEGs including 321 up-regulated and 524 down-regulated DEGs were obtained from gene profile 2. Accordingly, 255 DEGs including 67 up-regulated and 188 down-regulated DEGs were obtained from gene profile 3. (Table 3)

**Table 3:** The differentially expressed circRNAs and genes of the downloaded expression profiles.

	Up regulation	Down regulation	Total
**circRNA profile**	8	102	110
**gene profile 1**	456	536	991
**gene profile 2**	321	524	845
**gene profile 3**	67	188	255

### The ceRNA analysis for significantly differentially expressed circRNAs and pathway analysis for their related genes

**Fig 1:**
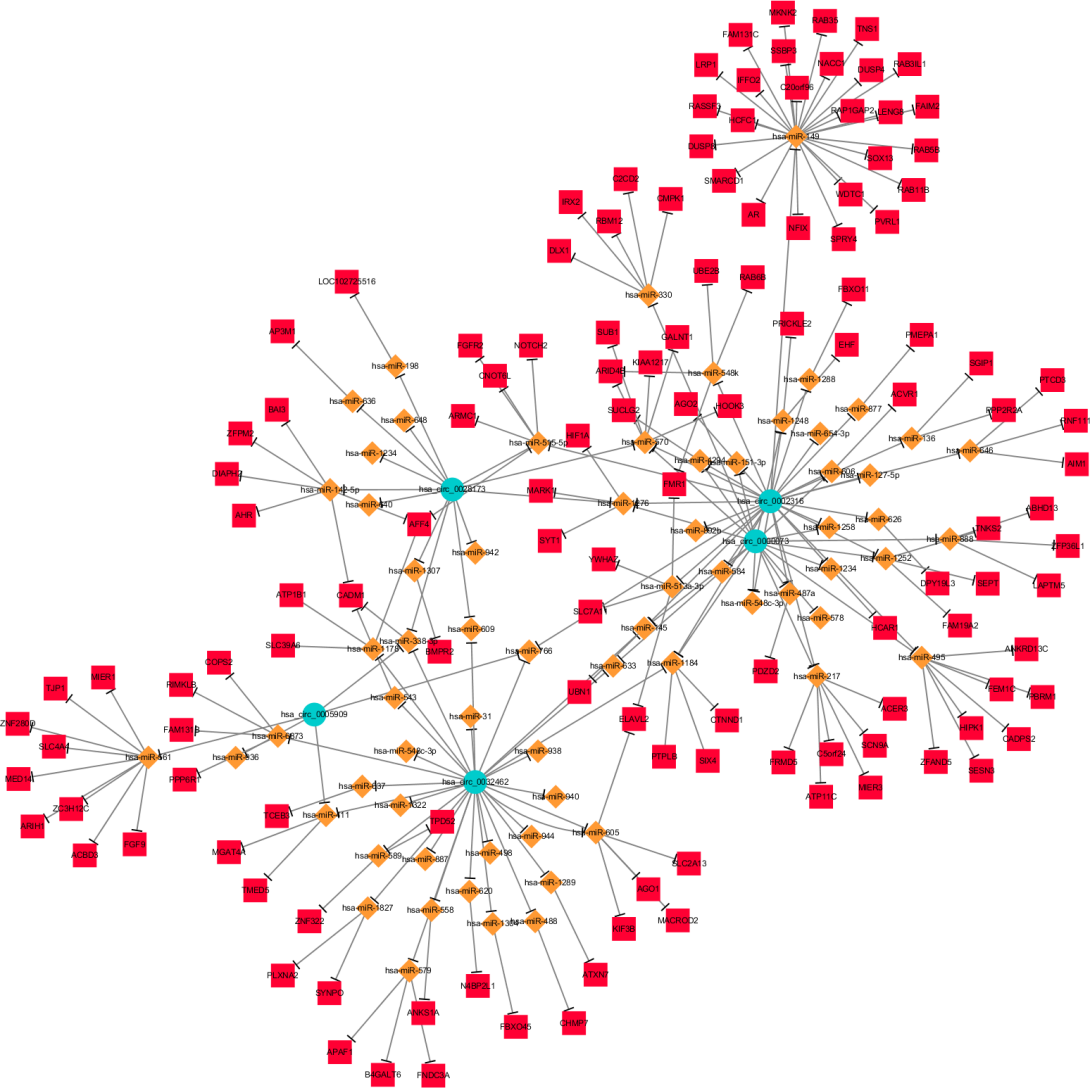
The ceRNA network of 5 most up-regulated circRNAs. The network consists of 5 circRNAs, 67 miRNAs and 127 mRNAs. In this network, the circRNA is marked by the circle, the shape of rhombus represents miRNA and the shape of rectangle represents mRNAs. Solid lines represent relationship between two nodes.

**Fig 2:**
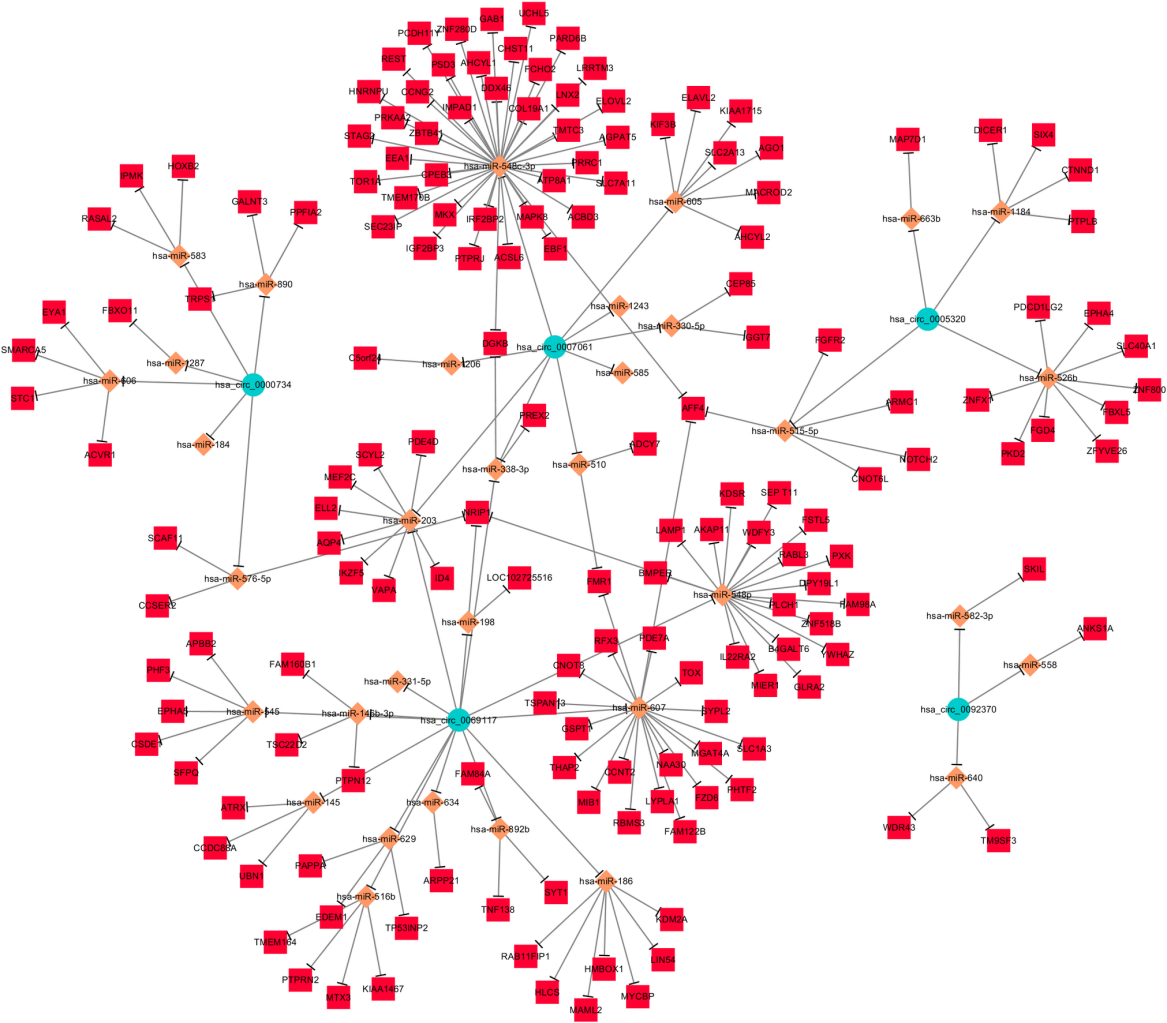
The ceRNA network of 5 most down-regulated circRNAs. The network consists of 5 circRNAs, 34 miRNAs and 158 mRNAs. In this network, the circRNA is marked by the circle, the shape of rhombus represents miRNA and the shape of rectangle represents mRNAs. Solid lines represent relationship between two nodes.

**Fig 3:**
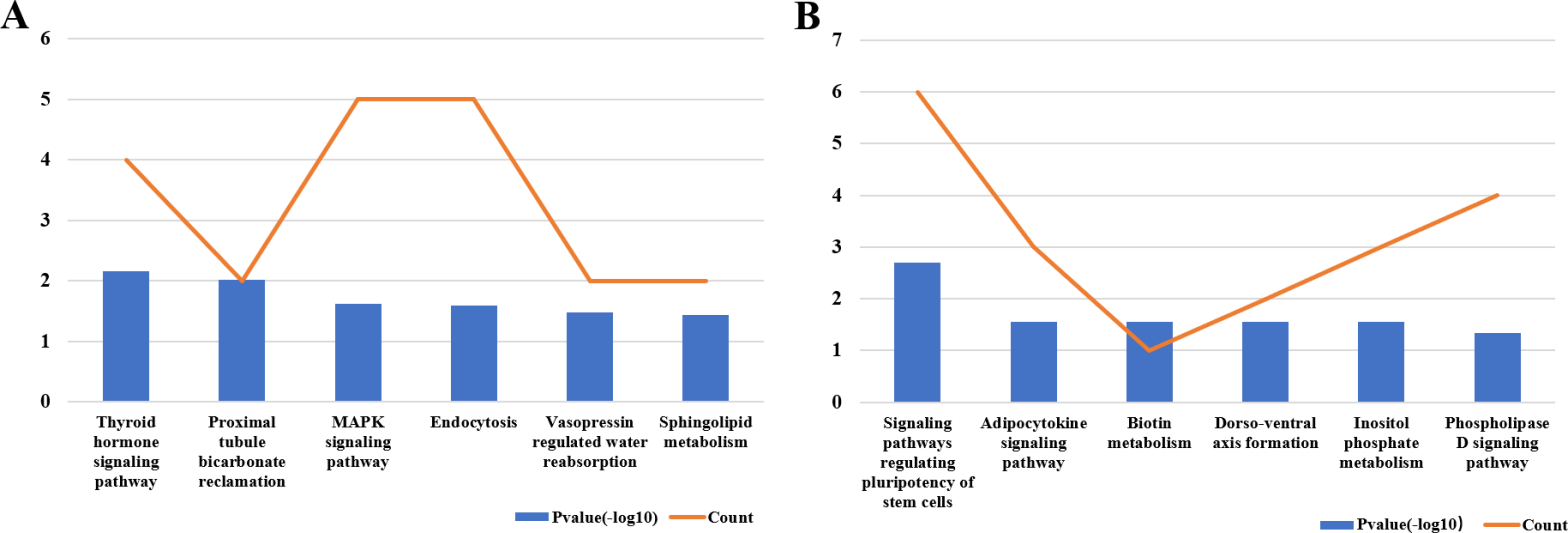
Pathway enrichment analysis of up- and down- regulated circRNAs related target genes. (A) The pathway enrichment of up-regulated circRNAs related target genes; (B) The pathway enrichment of down-regulated circRNAs related target genes.

Supposing that the most significantly differentially expressed circRNAs play important roles in OS through the proposed ceRNA mechanism, circRNA-miRNA- target gene networks of the 5 most up- and 5 most down-regulated circRNAs were constructed separately using Cytoscape. In the network of 5 most up-regulated circRNAs, 67 miRNAs ranked relatively higher, and 127 of the most likely target genes of these miRNAs were collected (Fig 1). Also 34 higher ranked miRNAs were found in the network of 5 most down regulated circRNAs, and 158 of the most likely target genes of these miRNAs were then collected (Fig 2). The pathway analysis showed that the up-regulated circRNAs related genes was meanly enriched in thyroid hormone signaling pathway, proximal tubule bicarbonate reclamation, MAPK signaling pathway, etc. (Fig 3A) The down-regulated circRNAs related genes were meanly enriched in signaling pathways regulating pluripotency of stem cells, adipocytokine, biotin metabolism etc. (Fig 3B)

### Pathway enrichment for DEGs

**Fig 4:**
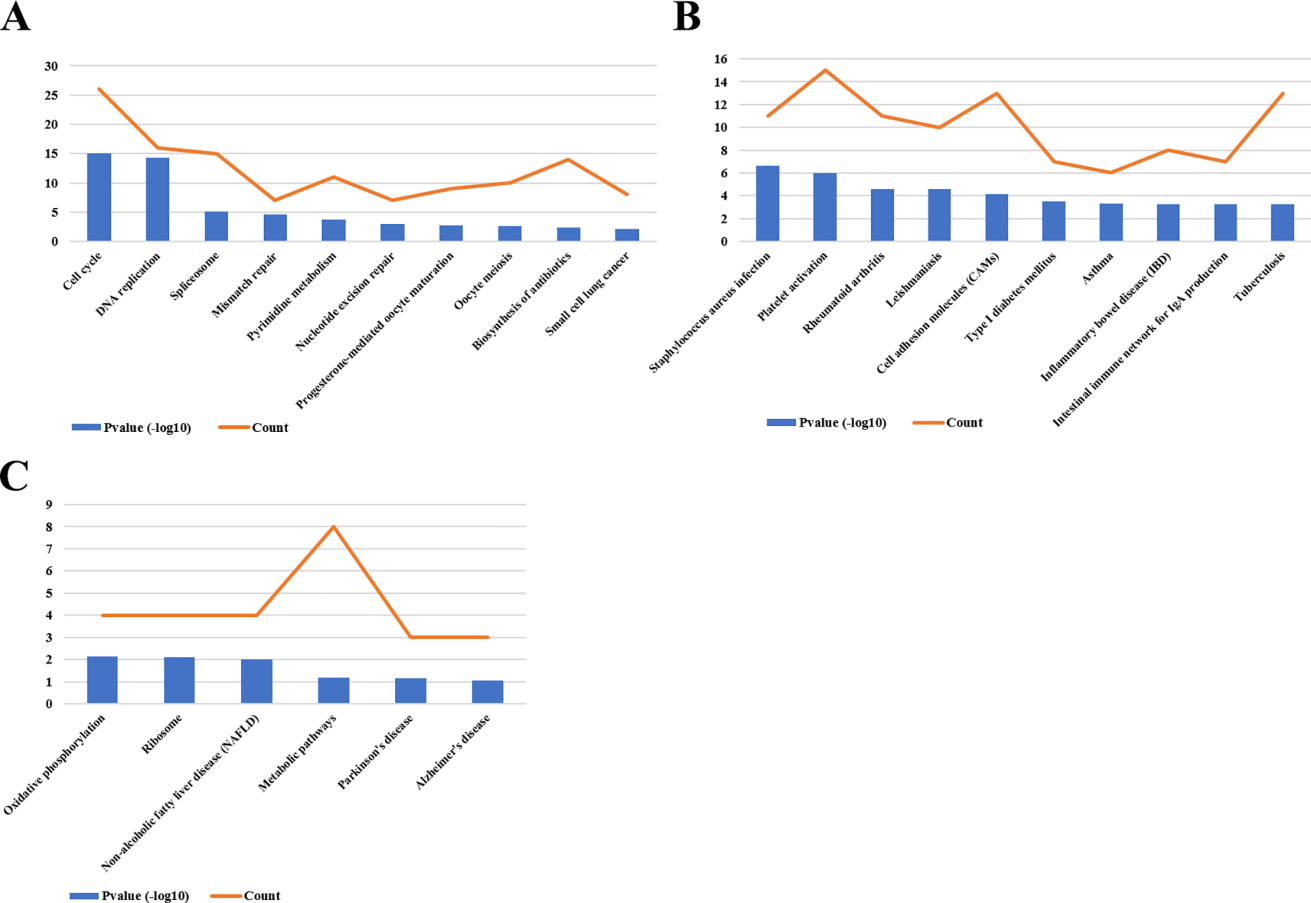
Kyoto Encyclopedia of Genes and Genomes (KEGG) pathway enrichment analysis of up-regulated DEGs from three gene expression profiles. (A) The most 10 significant pathways and the DEGs count involved in these pathways of up-regulated DEGs from gene profile 1. (B) The most 10 significant pathways and the DEGs count involved in these pathways of up-regulated DEGs from gene profile 2. (C) The significant pathways and the DEGs count involved in these pathways of up-regulated DEGs from gene profile 3.

**Fig 5:**
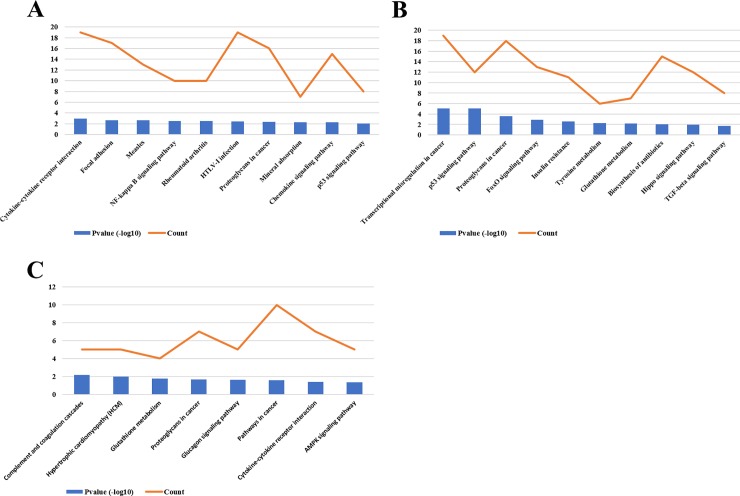
Kyoto Encyclopedia of Genes and Genomes (KEGG) pathway enrichment analysis of down-regulated DEGs from three gene expression profiles. (A) The most 10 significant pathways and the DEGs count involved in these pathways of down-regulated DEGs from gene profile 1. (B) The most 10 significant pathways and the DEGs count involved in these pathways of down-regulated DEGs from gene profile 2. (C) The significant pathways and the DEGs count involved in these pathways of down-regulated DEGs from gene profile 3.

KEGG pathway enrichment analysis was applied using DAVID for a further understanding of the differentially expressed genes function enrichment from three gene expression profiles. Based on the thresholds of P < 0.05, the pathway enrichment analysis revealed that the up-regulated DEGs of three gene profiles were significantly involved in 20, 31, 3 pathways separately. The down-regulated DEGs of three gene profiles were significantly involved in 25, 18, 8 pathways separately. The most significant pathways involved in the three gene expression profiles were listed in Figs 4 and 5.

### The co-expressed DEGs

**Fig 6:**
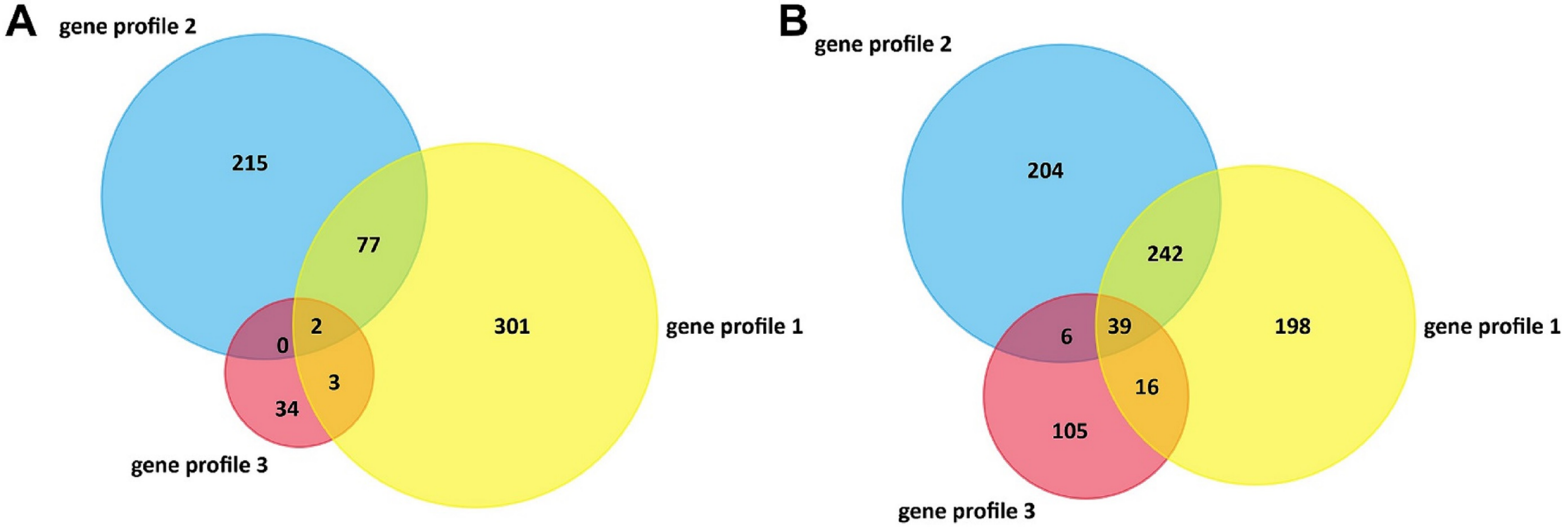
TheVenn analysis of up- and down-regulated DEGs. (A) The venn analysis of up-regulated DEGs showed there are 2 DEGs co-expressed in all three gene profiles and 80 DEGs co-expressed in any two gene profiles. (B) The venn analysis of down-regulated DEGs presented there are 39 DEGs were co-expressed in all three gene profiles and 264 DEGs were co-expressed in any two gene profiles.

The DEGs, up-regulated and down-regulated separately, were selected from three gene expression profiles and the co-expressed DEGs were generated using Venn analysis. There are 2(one is a pseudogene) co-expressed up-regulated DEGs (Fig 6A) and 39 down-regulated ones (Fig 6B) in all three gene profiles, and these DEGs were showed in Table 4. However, there were also 80 co-expressed up-regulated DEGs and 264 down-regulated ones between any two gene profiles.

**Table 4:** The co-expression DEGs of three gene expression profiles.

	Up-regulated DEGs	Down-regulated DEGs
**co-expression DEGs**	*CADM1*, *DNM1P46*	*IGFBP4*, *PTX3*, *SERPINE1*, *COL8A1*, *AOX1*, *MAOA*, *ANGPTL4*, *TRNP1*, *PLPP3*, *NPR3*, *SRGN*, *SORBS2*, *TAGLN*, *LPAR1*, *CCL20*, *CAV1*, *ANPEP*, *LHFP*, *TFPI*, *ITPRIP*, *PYGB*, *RGS4*, *ITGA5*, *FKBP5*, *EPS8*, *PLIN2*, *CXCL5*, *CLCF1*, *FAM129B*, *NR2F1*, *FN1*, *CD59*, *TGFBR2*, *PRR16*, *LOXL3*, *OLAH*, *TBC1D2*, *CYGB*, *PDE7B*

### PPI network of co-expressed DEGs

**Fig 7:**
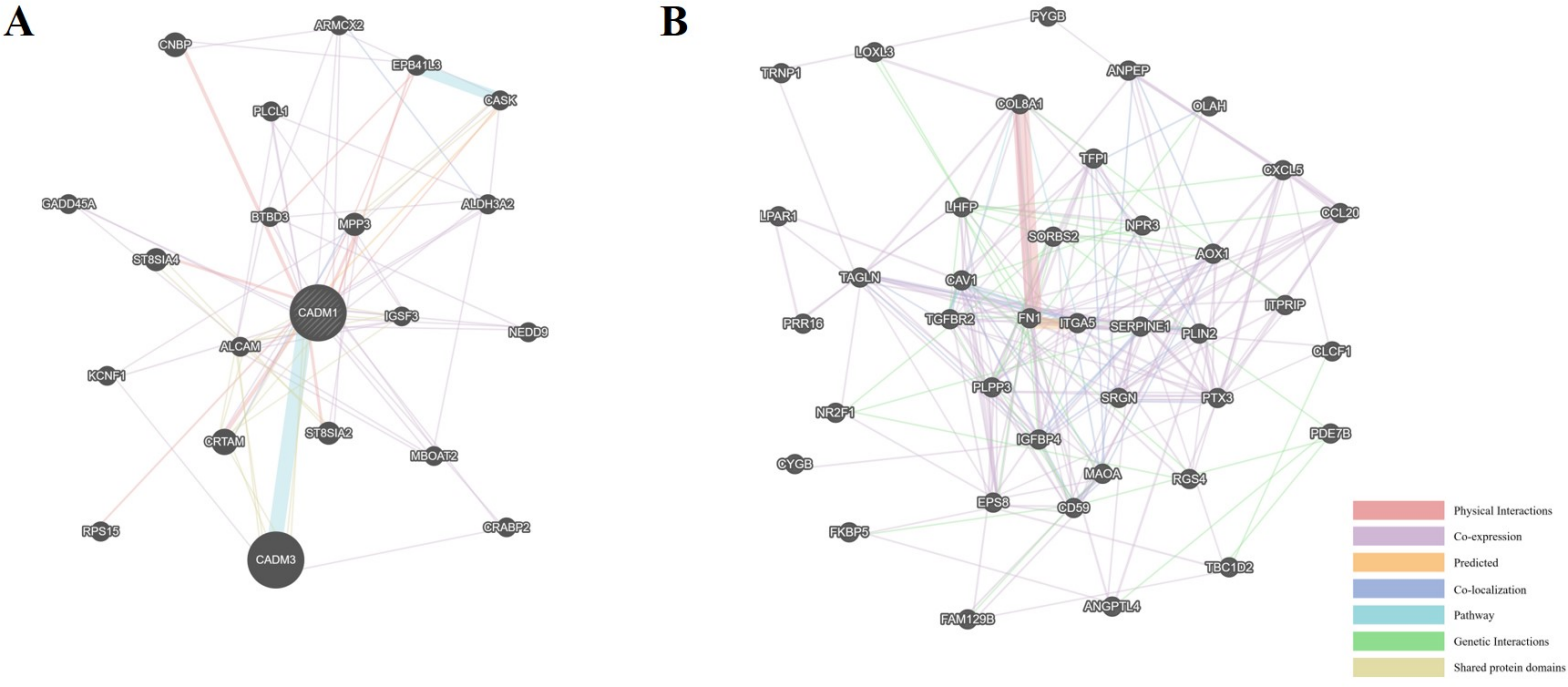
The protein-protein interaction (PPI) networks of up- and down-regulated DEGs which co-expressed in all three gene profiles. (A) The PPI network of 1 up-regulated DEG (CADM1) and proteins related to CADM1. (B) The PPI network of 39 down-regulated DEGs and their relationships.

Based on the information in the GeneMANIA protein query from public databases, we made the PPI network of 1 up-regulated gene and 39-downregulated genes which co-expressed by all three gene profiles. The genes related to the up-regulated DEG (CADM1) were associated with guanylate kinase activity, regulation of nature killer cell mediated cytotoxicity and cell-cell junction (Fig 7A). While the down-regulated DEGs and the genes related to them were associated with cell growth, differentiation, and platelet alpha granule (Fig 7B).

### The ceRNA network of CADM1 and hsa_circ_0032462, hsa_circ_0028173, hsa_circ_0005909

**Fig 8:**
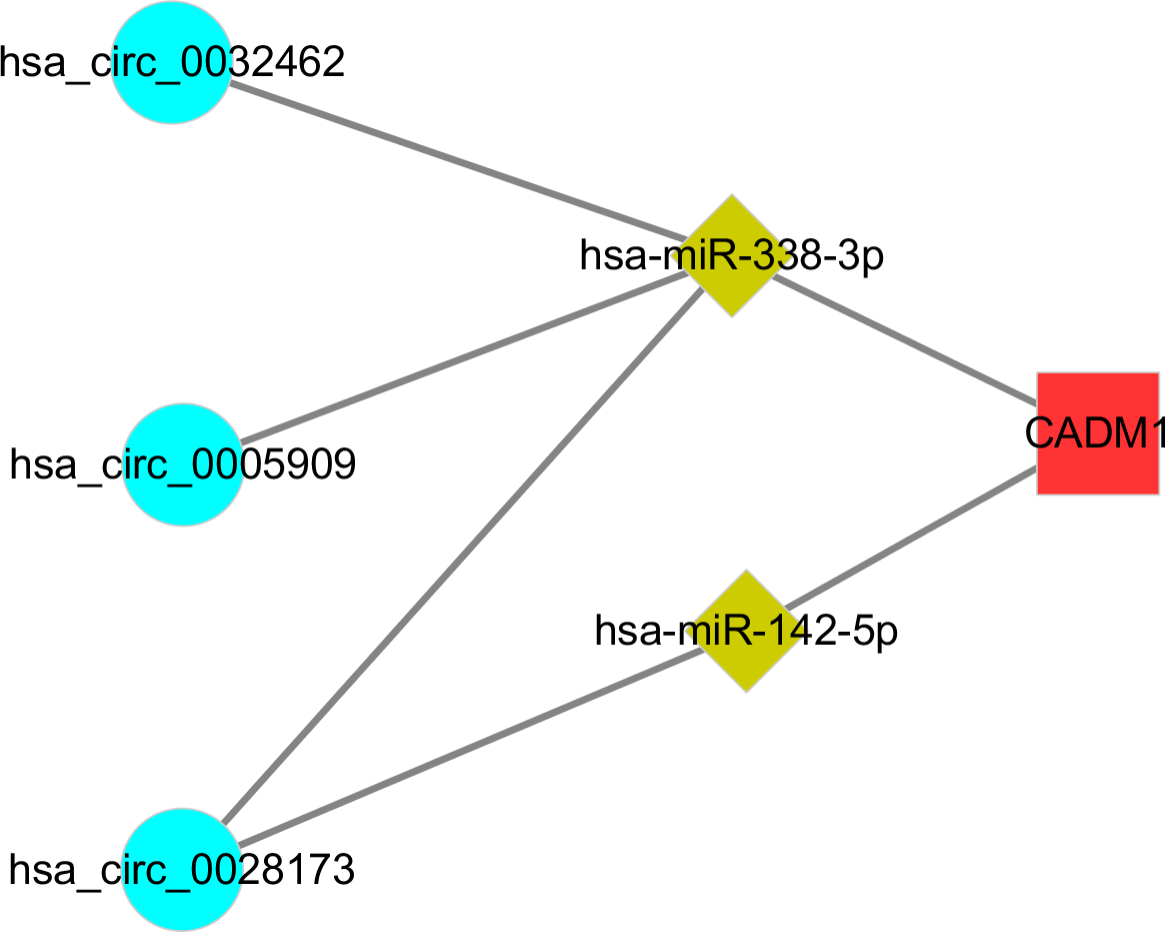
The circRNAs-miRNAs-CADM1 network.

**Fig 9:**
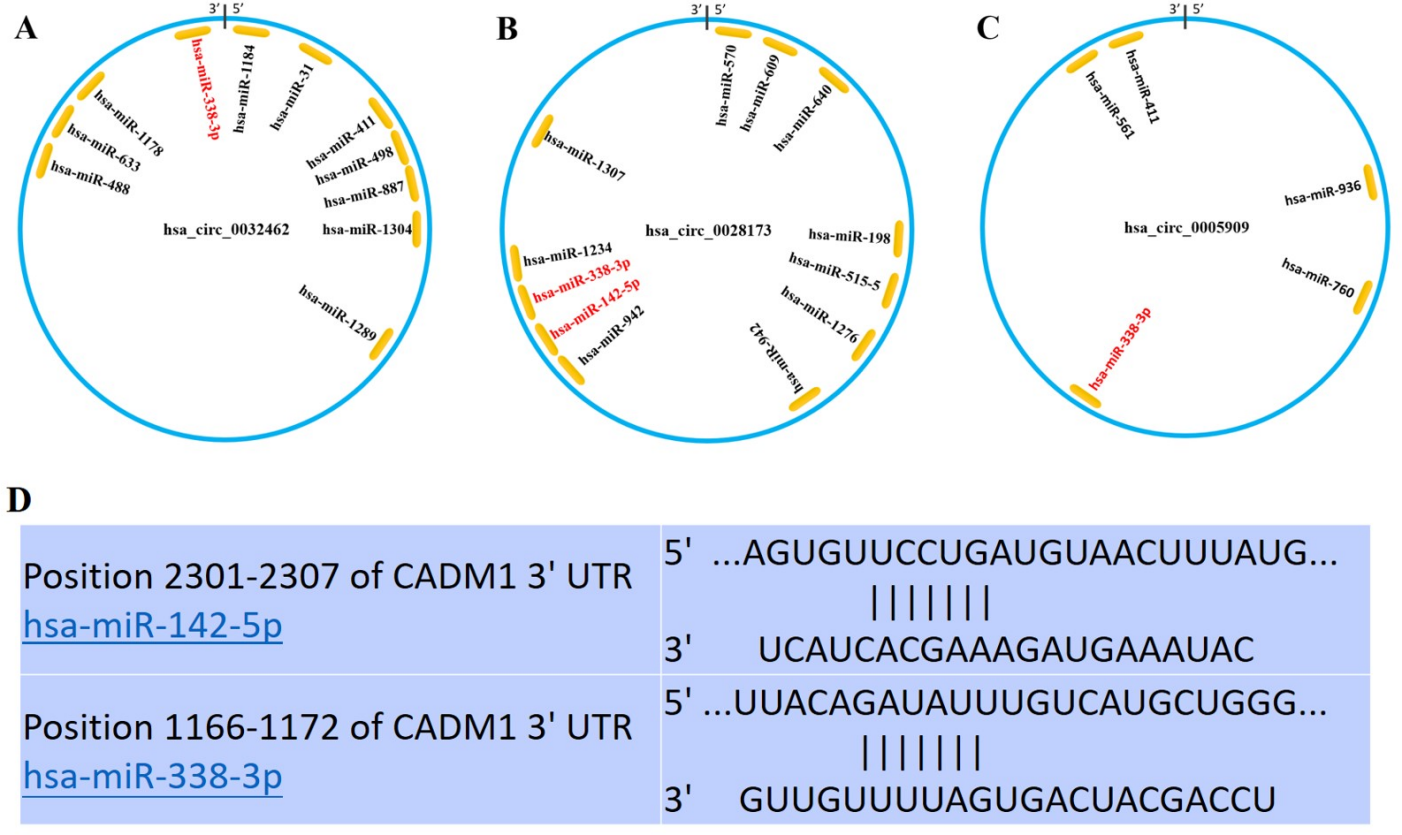
(A-C) Schematic models showing the putative binding sites for miRNAs and 3′UTR of three circRNAs. (D) Sequences within the CADM1 mRNA that is complementary to has-miR-142-5p and has-miR-338-3p were identified using publicly available algorithms.

The circRNA-miRNA-target gene networks were constructed using Cytoscape and several genes were found to be as the target genes. The Venn analysis generated 2 co-expressed up regulated DEGs (one is a pseudogene) and 39 down regulated ones in all three gene profiles. CADM1 was not only found in the ceRNA network, but also found as a co-expressed up-regulated DEG. Thus, we construct a ceRNA network including hsa_circ_0032462, hsa_circ_0028173, hsa_circ_0005909 and CADM1 (Fig 8). The circRNA hsa_circ_0032462 was predicted to regulate the expression of CADM1 by functioning as sponge of has-miR-338-3p. The circRNA hsa_circ_0028173 was predicted to regulate the expression of CADM1 by functioning as sponge of has-miR-142-5p and has-miR-338-3p. Accordingly, the circRNA hsa_circ_0005909 was predicted to regulate the expression of CADM1 by functioning as sponge of has-miR-338-3p. Schematic models were constructed to show the putative binding sites for miRNAs and 3′UTR of three circRNAs (Fig 9A–9C) and sequences within the CADM1 mRNA that is complementary to has-miR-142-5p and has-miR-338-3p were identified using publicly available algorithms(Fig 9D).

### Real-time qPCR validation and RNA interference

**Fig 10:**
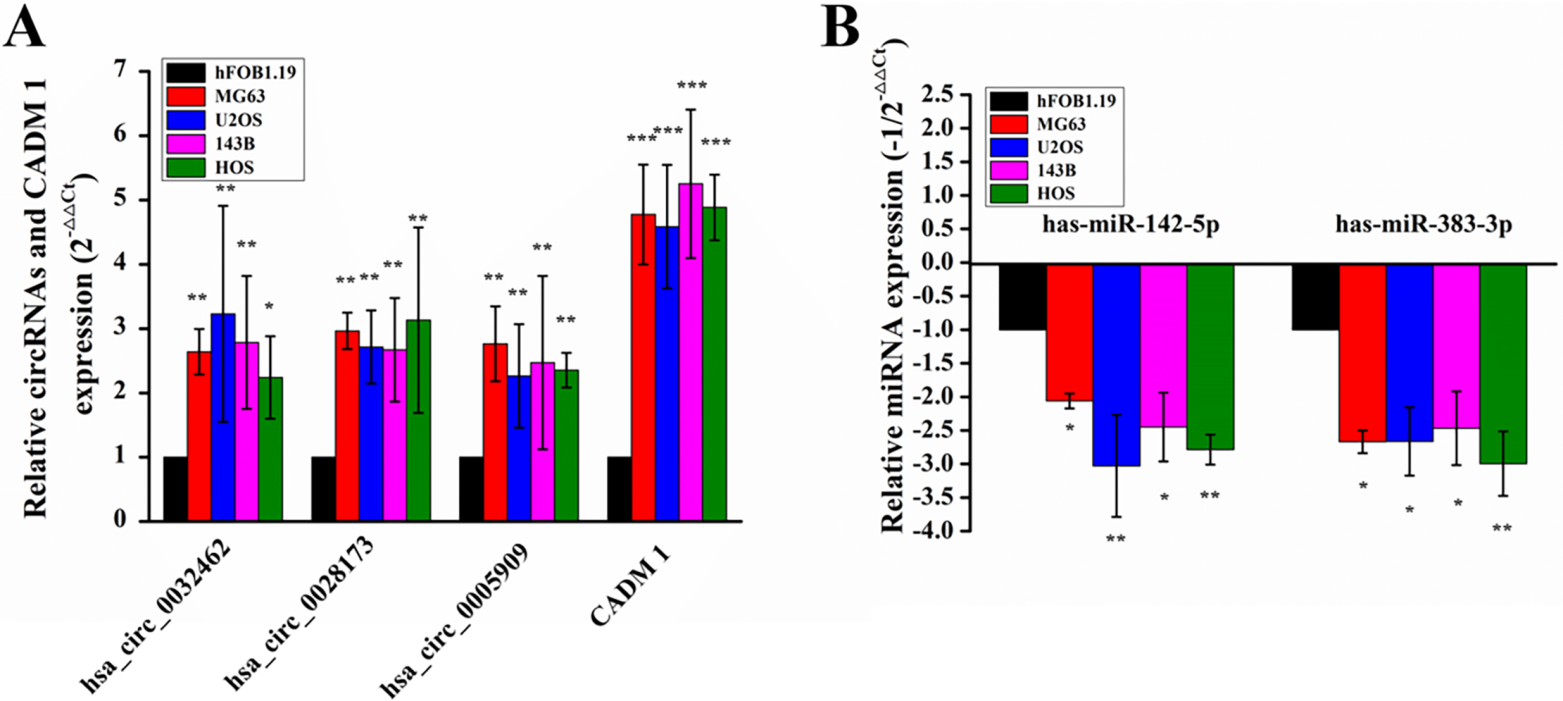
Validation of related circRNAs, miRNAs and CADM1 expression level in four human osteosarcoma cell lines (MG63, U2OS, 143B, HOS) and the human osteoblast cell line (hFOB1.19) using real-time qPCR. (A) Expression level of Circular RNAs hsa_circ_0032462, hsa_circ_0028173, hsa_circ_0005909 and CADM1 were significantly over-regulated in four osteosarcoma cell lines compared to osteoblast cell line (n = 3, * p<0.05, ** p<0.01, *** p<0.001). (B) Expression level of has-miR-142-5p and has-miR-383-3p were significantly down-regulated in four osteosarcoma cell lines compared to osteoblast cell line (n = 3, * p<0.05, ** p<0.01).

**Fig 11:**
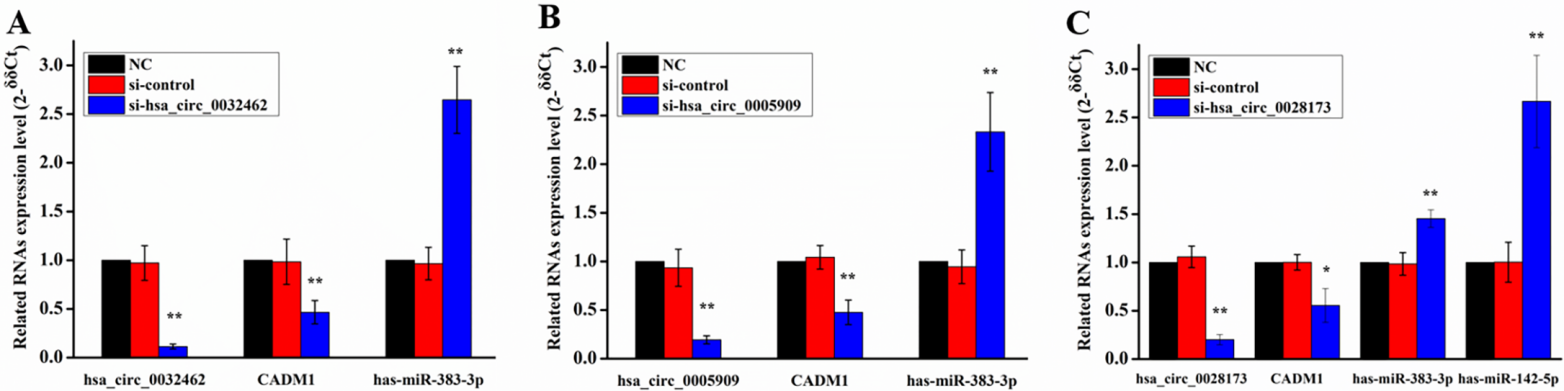
Knockdown of (A) circRNA_00032462, (B) circRNA_0005909 and (C) hsa_circ_0028173 can significantly inhibit expression of CADM1 in human MG63 cell line. (n = 3, * p<0.05, ** p<0.01).

We validated the expression of above RNAs using Real-time qPCR in four human osteosarcoma cell lines (MG63, U2OS, 143B, HOS) and the human osteoblast cell line (hFOB1.19). The expression of three circRNAs and CADM1 over-regulated significantly in four human osteosarcoma cell lines compared to osteoblast cell line (Fig 10A, p<0.05) and significant down-regulation could be seen in the has-miR-142-5p and has-miR-338-3p (Fig 10B, p<0.05). The siRNAs were used to knock down the expression levels of circRNAs and then detected the expression levels of CADM1 in MG63 cell lines. The expression levels of three circRNAs and CADM1 were found decreased significantly. However, the related miRNAs were found significantly over-expressed (Fig 11).

## Discussion

Although many previous studies have shown a number of genes were associated with the development and progression of OS [17]. The molecular mechanism of OS remains little known. However, some noncoding RNAs have been proved to play important roles in the pathogenesis of OS recently. LncRNA FENDRR was found to sensitize doxorubicin-resistance of osteosarcoma cells through down-regulating ABCB1 and ABCC1 [18]. Gaia Palmini et al. summarized many OS-related miRNAs (such as miR27, miR367, miR184, etc) in a review and indicted these biomolecules could provide a solution in the development of new therapies for OS [10]. Also, the circRNAs have been found differentially expressed in many diseases and some of them have been proved to play important roles in the pathogenesis of these diseases [19], [20]. Liu et al. has identified a comprehensive expression profile of circRNAs in osteosarcoma. However, the function of circRNAs in OS need more evidences[21].

In the present study, 7 OS samples (OS cell lines) and 1 control sample (osteoblast cell line) were included in the circRNAs microarray expression profile GSE96964. A total of 110 differentially expressed circRNAs were screened including 8 up-regulated circRNA and 102 down-regulated circRNAs. We also found 3 gene expression profiles of OS in order to find the DEGs in OS compared to osteoblast cells (Table 1).

The ceRNA hypothesis described how different types of coding and non-coding members of the transcriptome communicate with each other via miRNAs, competing for binding to miRNAs and then regulating the expression of each other to construct a complex post-transcriptional regulatory network [22]. Therefore, the ceRNA networks of 5 most up-regulated and 5 most down-regulated circRNAs were then constructed in order to predict their potential competing endogenous mRNAs. The results showed that 127 mRNAs may play as competing endogenous mRNAs of 5 most up-regulated circRNAs and 158 mRNAs may play as competing endogenous mRNAs of 5 most down-regulated circRNAs via miRNAs (Figs 1 and 2), which indicated that these 10 circRNAs may regulate the expression of these genes by competitively binding miRNAs. The pathway analysis showed lots of these circRNA-related genes are enriched in pathways which play important roles in the progression of OS (Fig 3), which also indicated that these differentially expressed circRNAs may play critical regulating roles in the progression of OS.

For a further understanding of DEGs function in three gene profiles, we also performed KEGG pathway analysis of these DEGs. The most significantly enriched pathways of up-regulated DEGs and down-regulated DEGs were showed in Figs 5 and 6. The result indicated that the up-regulated DEGs of three gene profiles significantly enriched in cell cycle, cell adhesion molecules (CAMs) and oxidative phosphorylation pathway. The down-regulated DEGs of three gene profiles significantly enriched in cytokine-cytokine receptor interaction pathway, p53 signaling pathway and proteoglycans in cancer pathway. Although these results are different to Fig 3, some studies have reported these pathways also play important role in the pathogenesis of OS [23–26].

DEGs seem to be important not only for their roles in OS associated pathways, but also for their potential as molecular markers of OS. Recent studies have revealed many molecular markers of OS, such as nephroblastoma overexpressed (CCN3), insulin like growth factor 2 (IGF2), U3 small nucleolar ribonucleoprotein (IMP3), miR-34 and so on [27–30]. Although the three gene expression profiles shared several pathways which have been reported to associate with the pathogenesis of OS. The DEGs of the three gene profiles are different owing to the different platforms and samples. Then the venn analysis was performed in order to find the co-expressed DEGs in the three gene expression profiles (Fig 6). The results demonstrated there are 2 (one is a pseudogene) co-expressed up-regulated DEGs and 39 down-regulated ones in all three gene profiles (Table 4). Further understanding of the protein function and protein-protein interaction of these DEGs will enable us to better understand the role of these DEGs in the pathogenesis of OS.

Fortunately, the PPI networks were constructed by GeneMANIA through either co-expression, co-localization, shared protein domains, predicted interactions, or genetic interactions to show the relationship of DEGs. The results indicated that the genes related to the up-regulated DEG (CADM1) were associated with guanylate kinase activity, regulation of nature killer cell mediated cytotoxicity and cell-cell junction. While the genes related to the down-regulated DEGs were associated with cell growth, differentiation, and platelet alpha granule. These functions were critical in the pathogenesis of OS [31–35]. We also noted that many of the co-expression genes have been prove to be involved in OS associated pathways in previous studies. The cell adhesion molecule 1 (CADM1) has been proved as a new osteoblastic cell adhesion molecule and a diagnostic marker for mouse OS [36]. Also, study have shown the differentially expressed insulin like growth factor binding protein 4 (IGFBP4) may be involved in MeCP2 gene-mediated proliferation and apoptosis in osteosarcoma cells [37]. The transgelin (TAGLN) plays a role in cell growth, differentiation, migration invasion and matrix remodeling by stabilizing the cytoskeleton through actin binding [38]. Caveolin-1 (CAV-1), which is an oncoprotein and a tumor suppressor, was demonstrated to be downregulated in OS cells, and overexpression of CAV-1 in human OS cells suppressed taxol resistance by attenuating PI3K-Akt-JNK-dependent autophagy [39]. The integrin subunit alpha 5 (ITGA5) is regulated by transforming growth factor β, and is involved in the adhesion of tumor cells to laminin in human OS cells [17]. C-X-C motif chemokine ligand 5 (CXCL5) has been proved to play a promoting role in OS cell migration and invasion in autocrine- and paracrine-dependent manners [40].

Interestingly, the CADM1 gene was not only found in the co-expressed up- regulated DEGs, but also as a competing endogenous mRNA of hsa_circ_0032462, hsa_circ_0028173, hsa_circ_0005909 by competitively binding to either has-miR-338-3p or has-miR-142-5p (Fig 8). Although these ncRNAs were not yet reported to be associated with osteosarcoma, previous study has shown that circRNAs hsa_circ_0032462 and hsa_circ_0005909 were differentially expressed in brain tissue, but the mechanism remains unknown [41]. The microRNAs has-miR-338-3p and has-miR-142-5p, which could be targeted by above circRNAs (Fig 8), have already been well studied for their inhibition effect on tumor cell growth in many diseases by targeting key genes[42–45]. Then we validated the expression of above circRNAs, miRNAs and CADM1 using Real-time qPCR in four human osteosarcoma cell lines and the human osteoblast cell line. These three circRNAs and CAMD1 all expressed with high level in osteosarcoma cell lines compared to osteoblast cell line, while the microRNAs has-miR-338-3p and has-miR-142-5p were found down-regulated (Fig 10). To further analyse the circRNA-miRNA-mRNA network, we used siRNA to knockdown these three circRNAs respectively. When the target circRNA were knocked-down, down-expressed level of CADM1 could be detected and, conversely, over-expressed levels of miRNA could be found. These results matched our predictions really well.

CADM1, the target gene in the map, is an intercellular adhesion molecule which belongs to the immunoglobulin superfamily [46]. Studies have demonstrated that the G/C-rich Sp1-binding sites of the CADM1 gene promoter could play a critical role in transactivation of this gene [47]. CADM1 was also found to localize on the lateral membrane of neighboring pancreatic islet cells and contributed to gap junctional communication [48]. Helvering et al. found that CADM1 clustered with collagens 1a1 and 1a2, osteocalcin, Sparc, and biglycan, which were already known bone formation activity genes [49]. Recent study has shown that CADM1 could regulate the G1/S transition and represses tumorigenicity through the Rb-E2F pathway in hepatocellular carcinoma [50]. CADM1 has also been found differentially expressed in melanocytic lesions and had the potential to contribute as an auxiliary tool to the differential diagnosis between nevi and melanomas [51]. It should be noted that Takao Inoue et al. identified CADM1 as a novel, easily detectable cell-membrane protein of OS. They also found that the majority of osteosarcomas were CADM1-positive, compaired with rarely positive of other soft tissue tumors outside osteosarcoma. These results indicated that CADM1 was useful in the differential diagnosis of OS [36]. In our study, the over expressed hsa_circ_0032462, hsa_circ_0028173, hsa_circ_0005909 were predicted to promote the expression of CADM1 via competitively binding miRNAs and then the over expressed CADM1 might participate in cell adhesion and enhanced the proliferation of OS cells, which promote the development of OS. These features proved CADM1 could not only be as a key molecular in the pathogenesis of OS, but also as a biomaker molecular, which may provide a new therapy strategy by suppressing CADM1 gene expression.

In present study, we performed a co-analysis of one circRNA and three mRNA microarray profiles to explore the potential roles of differentially expressed circRNAs in the pathogenesis of OS and we also validated the expression of three circRNAs, miRNAs and CADM1 using Real-time qPCR and RNA interference in human osteosarcoma cell lines. However, there were still some limitations. Only the most significantly expressed circRNA (5 up-regulated and 5 down-regulated) were studied by ceRNA analysis in our research and others remained unknown. Besides, the further experimental studies should be conducted to verify our findings in the future.

In summary, there were 110 circRNAs either over- or under-expressed in OS. Accordingly, 2 up regulated DEGs and 39 down regulated ones were found co-expressed in all three gene profiles of OS. By using bioinformatics analysis, we predicted over-expressed circRNAs circRNA_101391, circRNA_101139, circRNA_100413 may be potential regulator of the up-expressed gene CADM1. These over-expressed circRNAs are predicted to associate with the over expression of CADM1 via competitively binding miRNAs and promote the development of OS. The mechanism needs to be further confirmed by specific studies. Additionally, these important moleculars may possess the potential to be used as biomarkers, which may provide a new strategy in diagnosis and therapy of OS.

## Abbreviations

CADM1: cell adhesion molecule 1
ceRNA: competing endogenous RNA
circRNA: circular RNA
DEG: differentially expressed gene
GEO: Gene Expression Omnibus
MREs: miRNA response elements
OS: Osteosarcoma
PPI: protein-protein interaction
